# A practical tip for estimating skeletal maturation in children aged < 3 years using humeral ossification on chest radiographs: A retrospective study

**DOI:** 10.1007/s00431-026-06760-6

**Published:** 2026-01-29

**Authors:** Tomohiro Tsuru, Shota Inoue, Hiromi Edo, Shuichi Suzuki, Kohsuke Imai, Taiki Nozaki, Hiroshi Shinmoto

**Affiliations:** 1 Department of Pediatrics, National Defense Medical College, Saitama, Japan; 2 Department of Radiology, National Defense Medical College, 3-2 Namiki, Tokorozawashi, Saitama Japan

**Keywords:** Skeletal maturation, Humeral head ossification centre, Chest radiography, Paediatric growth assessment

## Abstract

Bone age assessment in children aged < 3 years is difficult owing to the limited visibility of carpal ossification centres. We aimed to evaluate skeletal maturation patterns in relation to chronological age using humeral ossification on chest radiographs. We retrospectively reviewed the chest radiographs of 187 children aged 0– < 3 years. Three readers independently measured the longitudinal diameter of the humeral head epiphyseal ossification centre. Interobserver agreement was assessed with the intraclass correlation coefficient (ICC). Pearson’s correlation and linear regression analyses were performed. Reference ranges (± 1 standard deviation [SD] and ± 2 SD) were established for six 6-month age groups. A strong positive correlation was observed between ossification centre diameter and age (r > 0.88 for all observers). The ICC was 0.96 (*p* < 0.001), indicating excellent interobserver agreement. Moreover, ± 2 SD reference ranges enabled the identification of deviations in skeletal maturation. A comparison by arm position (raised vs. lowered) revealed no significant difference (*p* = 0.454), supporting the robustness of the measurement. Additionally, simplified estimation formulas were proposed for practical clinical reference: the longitudinal diameter of the humeral head epiphyseal ossification centre (mm) = 0.5 × age in months + 4 for males and = 0.6 × age in months + 3 for females.

*Conclusion*: This study presents a simple, reproducible method using routine chest radiographs as a supplementary tool to assess skeletal maturation and to approximate chronological age in children aged < 3 years.

**Table:** 

**What is Known:** • *Assessment of skeletal maturity and bone age in children aged < 3 years is difficult owing to immature carpal ossification.* • *Traditional hand-based methods are often unreliable and require extra imaging.*	
**What is New:** • *A strong linear association between the longitudinal diameter of the humeral head ossification centre and chronological age was demonstrated in children aged < 3 years.* • *Sex-specific simplified estimation formulas and ± 2 SD reference ranges were established, allowing rapid assessment of skeletal maturation without additional imaging.*

**Supplementary Information:**

The online version contains supplementary material available at 10.1007/s00431-026-06760-6.

## Introduction

Bone age, as a reflection of skeletal maturation, is an essential clinical indicator for evaluating paediatric growth and detecting endocrine or nutritional abnormalities. Although classical methods, such as the Greulich and Pyle Atlas and Tanner–Whitehouse systems, have been widely used, these methods are well established for children aged ≥ 3 years and remain the standard approach for skeletal maturity assessment [1]. However, in infants and toddlers aged < 3 years, assessment of skeletal maturation using hand and wrist radiographs can be challenging in clinical practice. This is mainly because the number and size of carpal ossification centres are limited, and their appearance timing shows substantial interindividual variability, making accurate evaluation and standardised interpretation more difficult [2]. In newborns and infants aged < 1 year, ossification centres are often absent, and their appearance significantly varies among individuals. Furthermore, standardised reference data are scarce, making skeletal maturity assessment based on carpal bones even more challenging in this age group. Previous methods for bone age assessment have often been complex and have demonstrated low interobserver agreement, making them challenging to use in routine clinical settings.

In actual clinical practice, alternative approaches, such as plain radiographs of the knee—using methods, such as the Sontag or hemiskeleton technique—are sometimes used for children aged < 3 years [3–5]. However, these methods require additional imaging, result in increased radiation exposure, and can be more burdensome in terms of logistics and workflow.Given that the proximal humeral epiphysis is frequently captured in routine chest radiographs, we hypothesised that the longitudinal diameter of the humeral head ossification centre could serve as a practical and reproducible indicator of skeletal maturation and its relationship with chronological age in children aged < 3 years. This approach can leverage existing imaging data without additional radiation exposure or complex procedures. Moreover, by utilising routinely obtained chest radiographs, this method may help detect abnormal skeletal maturation or growth disorders at an early stage while avoiding additional imaging, thereby reducing radiation exposure and clinical workload. However, to date, no quantitative framework has been established to characterise skeletal maturation using humeral ossification centres visible on chest radiographs. Therefore, the aim of this study was to develop and evaluate a simple and reproducible quantitative method to assess skeletal maturation patterns and approximate chronological age in children under 3 years of age.

## Materials and Methods

### Study population and image selection

In this study, we included consecutive patients aged 0– < 3 years who underwent plain chest radiography at our hospital between January 2023 and December 2023. Eligible participants were required to have at least one chest radiograph obtained during the study period. When multiple chest radiographs were available for a participant during the study period, the most recent radiograph was selected for analysis.

### Inclusion and exclusion criteria

Patients meeting the study population criteria described above were included. The exclusion criteria were predefined as conditions likely to deviate from normal skeletal growth patterns, including prematurity (birth before 37 completed weeks of gestation) or low birth weight, congenital heart disease, genetic abnormalities, developmental disorders, suspected or confirmed child abuse, laryngomalacia, epilepsy or seizures, skeletal dysplasia, encephalopathy, and radiographs that were not suitable for evaluation. These criteria were applied before enrolment.

### Measurement

All chest radiographs were obtained using digital radiography systems from FUJIFILM Healthcare Corporation (Tokyo, Japan), including FUJIFILM DR BENEO, FUJIFILM DR CALNEO U, and FUJIFILM DR BENEO-Fx units installed in our institution. All images were acquired under standard paediatric chest radiography protocols with a fixed source-to-image distance. To verify measurement consistency, radiologic technologists performed calibration testing across the different units using a radiopaque ruler as part of a one-time quality assessment conducted specifically for this study. This assessment showed that the mean magnification difference among devices was 1.0275 (within approximately 3%), indicating negligible variation in image scaling.

Three readers with different levels of experience and backgrounds independently measured the longitudinal diameter of the ossification centre of the humeral head on chest radiography. All measurements were performed using the digital callipers integrated into our picture archiving and communication system (PACS). Window and level settings were standardised according to the institutional paediatric chest radiography protocol. Magnification correction was not required because all images were acquired with a fixed source-to-image distance and device-specific magnification variability was confirmed to be negligible through calibration testing, as described above.

**Fig. 1:**
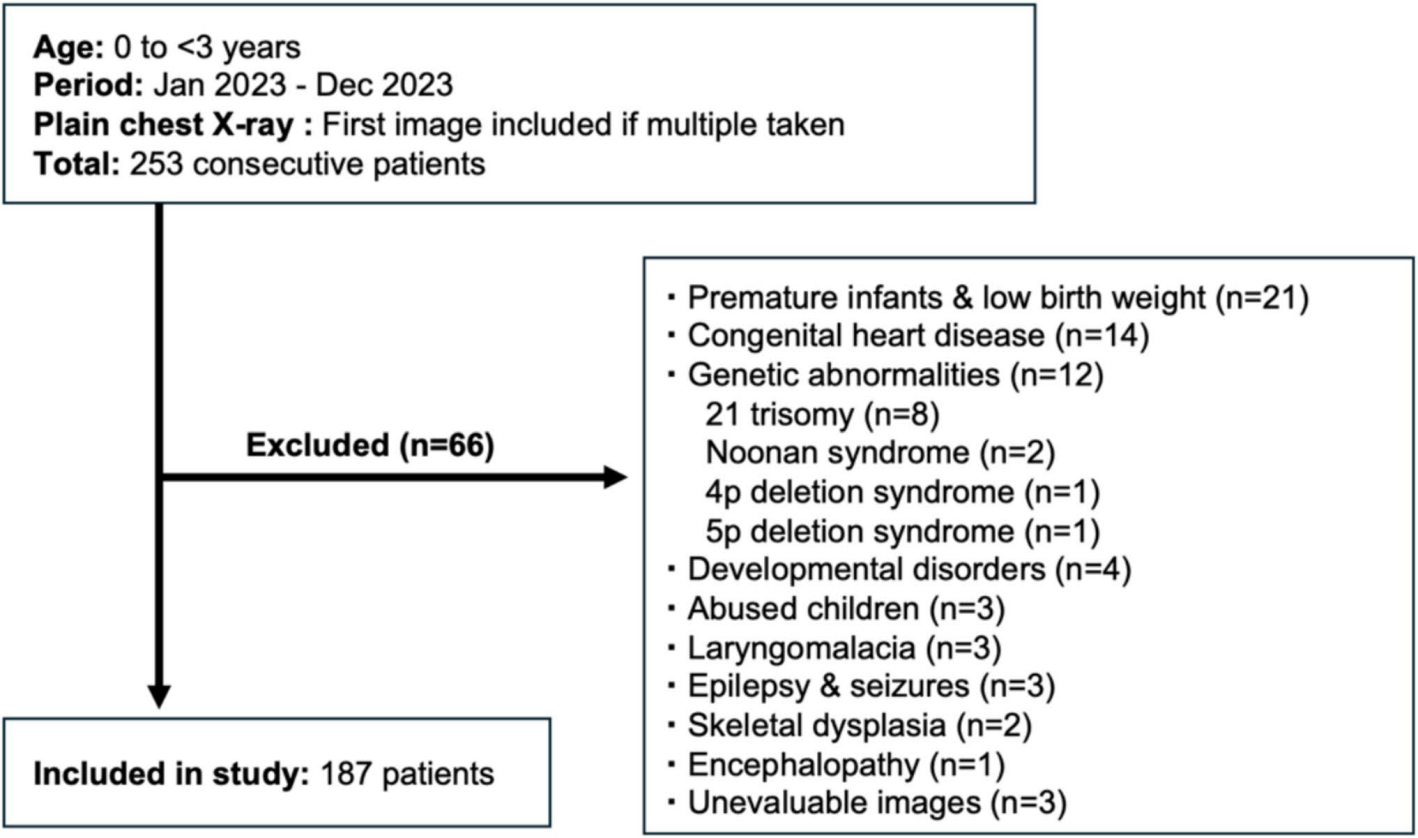
Patient selection and exclusion criteria

Each reader performed the measurements independently and was blinded to participants’ chronological age. The readers were also blinded to each other’s measurements and did not have access to the measurements made by the other observers during the study period. All readers initially performed measurements independently while blinded to participants’ age and to each other’s results. After completion of the initial measurements, the readers jointly reviewed the images solely to confirm that the same humeral head ossification centre had been selected as the measurement target. In cases where different ossification centres had been measured, each reader independently re-measured the correct ossification centre without access to the other readers’ measurement values. No harmonisation or averaging of measurements was performed. The longitudinal diameter was defined as the longest measurable axis of the humeral head ossification centre visible on the radiograph. Cases in which the humeral head ossification centre was not visually identifiable were not treated as missing data but were assigned a value of 0 mm, reflecting the biological absence of a measurable ossification centre rather than data unavailability (Fig. 1).

**Fig. 2:**
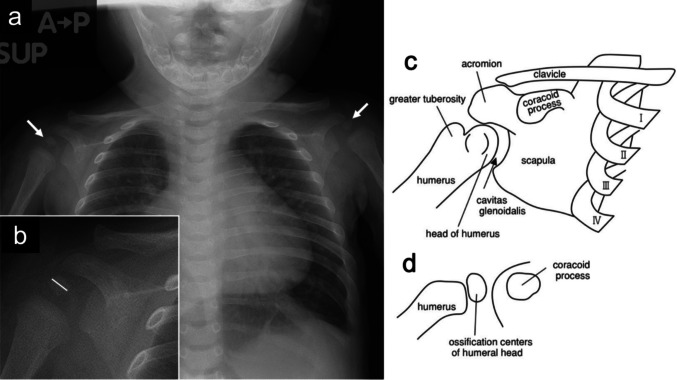
Typical example of a 3-month-old female infant with arms lowered. (**a**) A plain chest radiograph showing ossification centres in the bilateral humeral heads (white arrows).. (**b**) Magnified view of the right humeral head from (a). The white line indicates the measurement axis for the long diameter of the ossification centre of the right humeral head.. (**c**) Schematic of the right shoulder joint in adulthood.. (**d**) Schematic of the right shoulder joint in a 3-month-old infant.. The schematic illustrations were created by the authors for this study

**Fig. 3:**
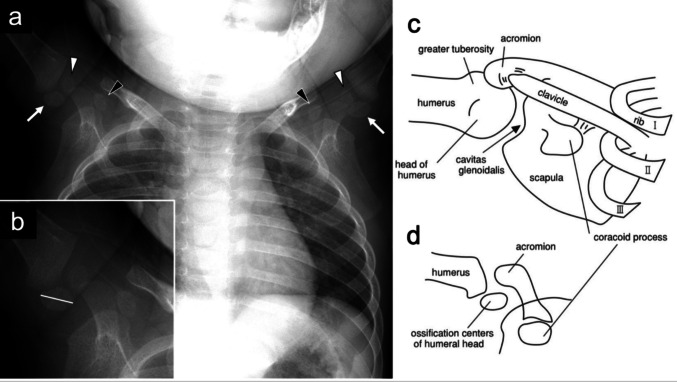
Typical example of a 7-month-old female infant with arms raised. (**a**) A plain chest radiograph showing ossification centres in the bilateral humeral heads (white arrows) and greater tuberosities (white arrowheads). The black arrowhead indicates the ossification centre of the coracoid process, which might easily confuse with the ossification centre of the humeral head.. (**b**) Magnified view of the right humeral head from (**a**). The white line represents the measurement axis for the long diameter of the ossification centre in the right humeral head.. (**c**) Schematic of the right shoulder joint in adulthood.. (**d**) Schematic of the right shoulder joint in a 7-month-old infant.. The schematic illustrations were created by the authors for this study

To assess interobserver agreement, measurements were compared among the three readers. Reader A had 3 years of postgraduate clinical experience, Reader B was a paediatrician with 6 years of postgraduate clinical experience, and Reader C was a board-certified radiologist with 16 years of postgraduate clinical experience. In cases where there was uncertainty regarding the identification of the ossification centre of the humeral head, the three physicians held a discussion to reach a consensus on the ossification centre, and each subsequently performed the measurements individually. Additionally, the presence or absence of the epiphyseal nucleus in the humeral head was evaluated. Furthermore, when multiple ossification centres were present at the proximal humeral head, the longest diameter of the largest ossification centre was measured. The ossification centres on the left and right sides were measured, and the larger value was adopted as the longitudinal diameter. If only one side could be measured, the diameter of the ossification centre on the measurable side was used. Measurements of the longitudinal diameter were analysed separately for each reader, and no post hoc adjustment or correction was applied to reconcile differences in measured values. For correlation analyses, linear regression modelling, and the construction of reference ranges, the measurements obtained by each reader were used independently. In contrast, consensus-based decisions were applied only for qualitative assessments, including identification of the target ossification centre and classification of arm position. For analyses requiring a single representative value per participant—specifically the arm-position comparison—the mean of the three readers’ measurements was used. Using this approach, no individual-level data were missing, as all participants possessed at least one measurable ossification center of the humeral head. The ossification centre was identified and measured according to the arm position, as shown in Fig. 2. Chest radiographs were obtained with the patient’s arms in either the raised (Fig. 2) or lowered position (Fig. 3), depending on the case. For analysis, arm position was classified as either raised or lowered, based on whether the arms were positioned above or below the horizontal plane on the radiograph.

### Visibility analysis of the humeral head ossification centre

To evaluate early developmental changes, the number and percentage of epiphyseal ossification centres observed in the humeral head were analysed based on age groups at 3-month intervals and stratified by sex (Online Resource 1; Table S1).

This analysis was conducted separately from the main 6-month grouping used for the regression and reference range analyses to better capture the rapid morphological changes occurring during infancy.

### Statistical analyses

Statistical analyses were performed using the statistical software R (4.1.1) [6].

### Normality assessment and descriptive statistics

The p-values of the Shapiro–Wilk normality test were calculated to assess the distribution of the longitudinal diameters. Scatterplots were created with age in months on the x-axis and the larger of the left or right longitudinal diameters on the y-axis. Descriptive statistics (mean, standard deviation, ± 1 SD and ± 2 SD ranges) were computed for each 6-month age group.

### Correlation analysis

Pearson’s correlation coefficients were calculated to evaluate the relationship between chronological age and the longitudinal diameter of the humeral head ossification centre.

### Regression modelling

Linearity was assessed by visual inspection of scatterplots and by the strength of the linear association, as reflected by high coefficients of determination (R^2^), which ranged from 0.818 to 0.841 for males and from 0.782 to 0.842 for females across readers. Given the strong linear relationships observed and the clinical preference for simple, interpretable models, linear regression was considered appropriate for this analysis. Linear regression analyses were performed to assess the association between age and ossification centre diameter. Regression coefficients, 95% confidence intervals, and coefficients of determination (R^2^) were calculated for each reader. To assess the assumption of homoscedasticity in the linear regression models, heteroscedasticity of the residuals was evaluated using the Breusch–Pagan test. This assessment was performed using the measurements obtained by Reader A as a representative observer, separately for males and females. Visual inspection of residual plots and scatterplots indicated similar distribution patterns and regression trends across the other readers, with comparable regression coefficients, as illustrated in Fig. 5.

### Interobserver reliability

Interobserver agreement among the three readers was evaluated using the intraclass correlation coefficient (ICC), calculated with a two-way random-effects model under the single-rater assumption, assessing absolute agreement among measurements [7].

### Visibility analysis

For early developmental evaluation, visibility of the ossification centres was analysed in 3-month age groups stratified by sex (Online Resource 1; Table S1).

### Arm-position effect analysis

For the arm-position analysis, the mean of the three readers’ measurements was used to reduce reader-related measurement variability and to isolate the effect of arm positioning itself. Reader-specific measurements were used in the regression analyses, as described above, to preserve inter-reader variability and reflect real-world measurement conditions. To examine whether arm position influenced measurement values, participants were categorised into raised and lowered arm-position groups. For each participant, the mean diameter was calculated by averaging the measurements from the three readers. A two-sample t-test assuming equal variances was performed to compare the mean diameters between the two groups.

## Results

### Participant selection and characteristics

A total of 253 children were initially screened for eligibility. Of these, 66 were excluded based on the predefined exclusion criteria, resulting in 187 participants in the final analysis (Fig. 1).

The indications for chest radiography were fever screening (*n* = 117), cardiac screening (*n* = 10), congenital evaluations (*n* = 8), child abuse screening (*n* = 2), other clinical presentations such as seizures, vomiting, wheezing, or suspected foreign-body ingestion (*n* = 48), and follow-up imaging for pneumonia or atelectasis (*n* = 2).

Demographic characteristics—including age distribution, sex, arm position during imaging, visibility of ossification centres, and imaging indications—are summarised in Table 1.

**Table 1:** Participant characteristics. Summary of demographic and imaging characteristics of the study population, including age distribution, sex, arm position during imaging, visibility of humeral head ossification centres, and clinical indications for chest radiography

Characteristic	Value
Number of participants, n	**187**
Age (months), mean ± SD/median [IQR]	15.1 ± 10.3/15.3 [17.0]
Sex, n (%)	
Male	113 (60.4%)
Female	74 (39.6%)
Arm position during imaging, n (%)	
Raised	154 (82.4%)
Lowered	33 (17.6%)
Visibility of humeral head ossification centre, n (%)	
Visible	179 (95.7%)
Not visible	8 (4.3%)
Indications for chest radiography, n (%)	
Fever screening	117 (62.6%)
Cardiac screening	10 (5.3%)
Congenital evaluation	8 (4.3%)
Child abuse screening	2 (1.1%)
Other clinical presentations*	48 (25.7%)
Follow-up for pneumonia/atelectasis	2 (1.1%)

^*^ Other clinical presentations include seizures, vomiting, wheezing, and suspected foreign-body ingestion.

### Visibility of the humeral head ossification centres

Ossification centres of the humeral head were visible in nearly all children aged 3 months or older.

In infants aged 0–2 months, bilateral ossification centres were not visible in 8 of 187 participants (4.3%; 5 males and 3 females). In these cases, the longitudinal diameter was defined as 0 mm for analysis.

In all other age groups, at least one ossification centre was visible in every participant.

### Interobserver reliability

Measurements of the longitudinal diameter demonstrated excellent interobserver agreement, with an intraclass correlation coefficient (ICC) of 0.96 (p < 0.001).

### Correlation between age and ossification centre length

**Fig. 4:**
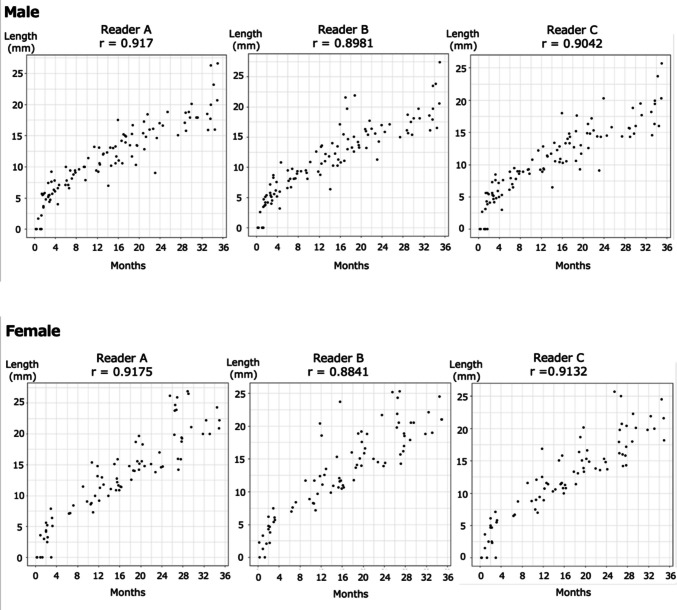
Correlation between age in months and ossification centre length of the humeral head in males and females. Scatterplots showing the correlation between age in months and the longitudinal diameter of the epiphyseal ossification centres of the humeral head measured independently by three readers (Readers A, B, and C) for both males and females. The Pearson correlation coefficients (r) for males were as follows: r = 0.917 (Reader A), r = 0.898 (Reader B), and r = 0.904 Reader C), and for females, r = 0.917 (Reader A), r = 0.884 (Reader B), and r = 0.913 (Reader C)

Scatterplots (Fig. 4) demonstrated a strong positive correlation between chronological age and ossification centre diameter across all three readers.

For males, Pearson correlation coefficients were 0.917 (Reader A), 0.898 (Reader B), and 0.904 (Reader C); for females, coefficients were 0.917, 0.884, and 0.913, respectively.

### Linear regression modelling

**Fig. 5:**
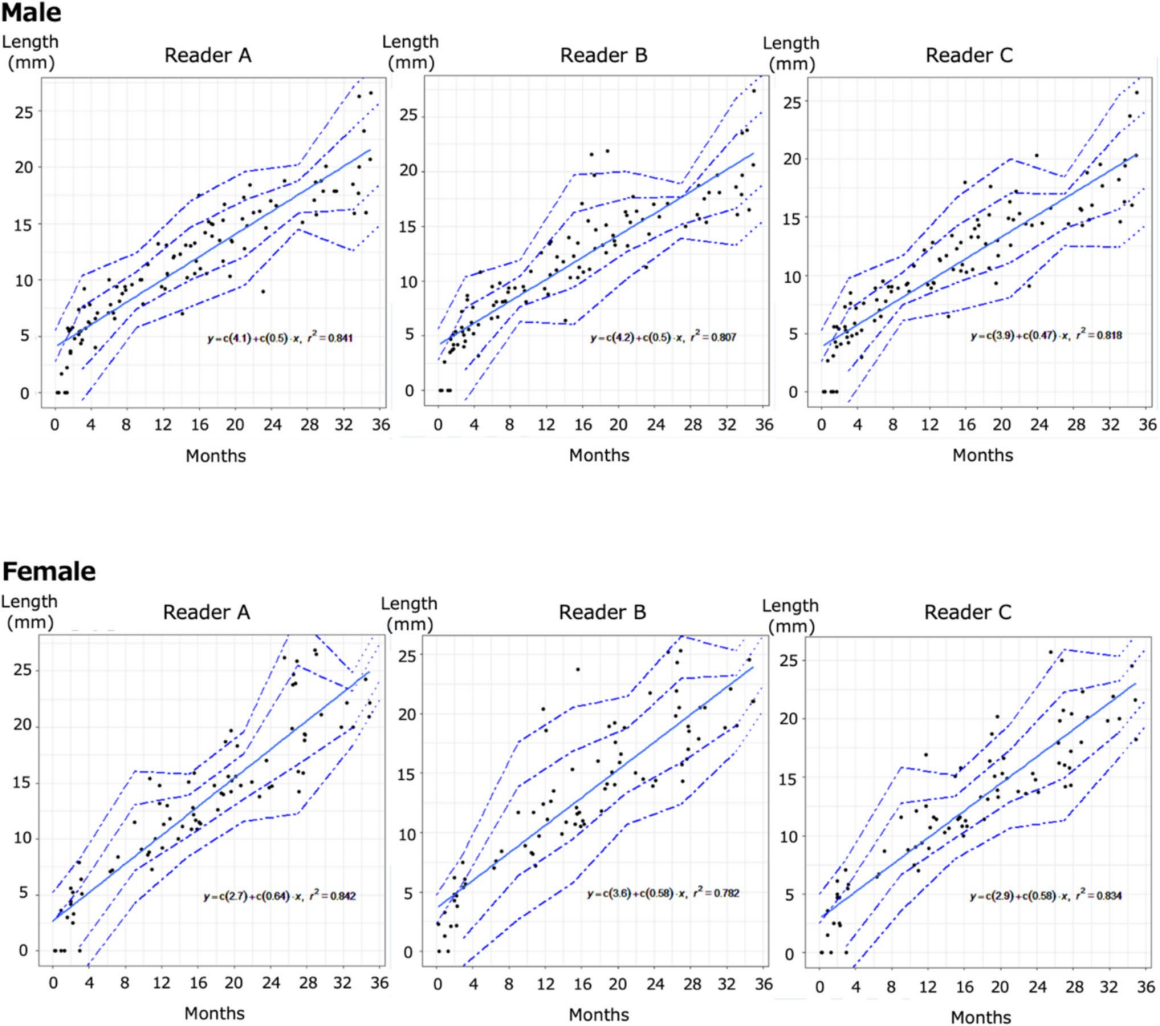
Linear approximation of longitudinal diameter of epiphyseal ossification centres in the humeral head for males and females. Linear approximations based on scatterplots of the longitudinal diameter of the epiphyseal ossification centres of the humeral head for both males and females. The solid lines represent the linear approximation, and the dotted lines represent ± 1 standard deviation (SD) and ± 2 SD, calculated from the sample mean and SD for six age groups at 6-month intervals. Data beyond 33 months were estimated using the approximation formula and sample SD. The coefficients of determination (R^2^) are as follows: and for males, R^2^ = 0.841 (Reader A), R^2^ = 0.807 (Reader B), and R^2^ = 0.818 (Reader C); for females, R^2^ = 0.842 (Reader A), R^2^ = 0.782 (Reader B), and R.^2^ = 0.834 (Reader C)

Linear regression analysis further confirmed a strong association between age and ossification centre diameter (Fig. 5).

The regression coefficients with 95% confidence intervals (CI) for males and females were as follows:

Reader A: 1.68 (95% CI: 1.54–1.82) for males; 1.32 (95% CI: 1.19–1.44) for females.

Reader B: 1.61 (95% CI: 1.46–1.77) for males; 1.34 (95% CI: 1.19–1.50) for females.

Reader C: 1.73 (95% CI: 1.57–1.89) for males; 1.45 (95% CI: 1.30–1.59) for females.

The coefficients of determination (R^2^) ranged from 0.841 to 0.818 for males and from 0.842 to 0.782 for females.

The full sex-specific regression coefficients for each reader, including slopes, intercepts, and 95% confidence intervals, are provided in Online Resource 2 (Table S2).

The Breusch–Pagan test indicated no significant heteroscedasticity in males (*p* = 0.0838), whereas evidence of heteroscedasticity was observed in females (*p* = 0.0061).

### Simplified estimation formulas

To facilitate clinical application, simplified estimation formulae were generated from the average slopes and intercepts of the three regression models:

For males:

Longitudinal diameter (mm) = 0.5 × age in months + 4.

For females:

Longitudinal diameter (mm) = 0.6 × age in months + 3.

Inverse equations for estimating chronological age from measured diameter were also obtained:

For males:

Age (months) = 2 × (diameter in mm − 4).

For females:

Age (months) = 1.7 × (diameter in mm − 3).

Clinical implications of these simplified formulae are described in the Discussion.

These simplified estimation formulas are summarised in Online Resource 3 (Table S3).

### Age-group reference ranges

Age was stratified into six 6-month groups (0–6, 6–12, 12–18, 18–24, 24–30, and 30–36 months).

For each group, the mean longitudinal diameter and corresponding ± 1 SD and ± 2 SD reference ranges were calculated using the average of the three readers’ measurements (Table 2).

**Table 2:** Average and standard deviations (SDs) (± 1 SD and ± 2 SD) of ossification centre lengths based on 6-month age groups

Male				
Age Group (Months)	n	Average (mm)*	−1SD (mm)*	−2SD (mm)*
0- 6	31	4.7	1.9	N.A
6–12	15	9.1	7.6	6.1
12–18	22	12.3	9.6	6.9
18–24	17	14.6	11.9	9.3
24–30	8	16.4	15.0	13.7
30–36	12	19.7	16.3	12.9
Female				
Age Group (Months)	n	Average (mm)*	−1SD (mm)*	−2SD (mm)*
0- 6	17	3.2	0.7	N.A
6–12	12	10.0	6.7	3.5
12–18	16	12.3	9.8	7.4
18–24	15	15.6	13.3	11.0
24–30	16	19.7	15.8	12.0
30–36	6	21.2	19.2	17.3

^*^The average, –1 SD, and –2 SD values were calculated based on the mean measurements obtained from three physicians

The table presents the average, –1 SD, and –2 SD values of ossification centre lengths measured in the proximal humerus of infants and young children, calculated from measurements taken by three physicians. For the 0–6 month group, –2 SD values were not calculated because they would result in values ≤ 0. Although all individual measurements were included in the analyses, the 30–36-month age group was sparsely represented; therefore, summary values for this age group were derived from the regression model rather than directly calculated from observed group means

Values for the 30–36-month group were estimated using the regression model, as this age range was not fully represented in the sample.

### Arm-position effect analysis

The comparison between raised and lowered arm-position groups showed no significant difference in the measured longitudinal diameter (*p* = 0.454), indicating that arm position did not substantially influence measurement values. The distribution of measurements by arm position is additionally illustrated in Online Resource 3 (Fig. S1).

## Discussion

To the best of our knowledge, this study is the first to establish a quantitative framework for assessing skeletal maturation in children aged < 3 years using the longitudinal diameter of the humeral head ossification centre, which is visible on chest radiographs. Our findings demonstrate a strong linear relationship with chronological age and excellent interobserver reliability, offering a feasible and non-invasive method for early skeletal assessment.

The proximal humeral epiphysis does not develop from a single ossification centre but rather through the fusion of smaller centres in the humeral head, greater tubercle, and lesser tubercle. Individual variability is present in the timing of appearance of these centres; for example, the humeral head epiphysis appears between 37 weeks of gestation and 6 months of age and the greater tubercle between 2 months and 2.5 years [8]. Typically, one ossification centre is visible at approximately 6 months, two by 1 year, and three by 2 years, which fuse into a single centre by the age of 4 years. This developmental pattern provides a biological rationale for considering the humeral head ossification centre as a marker of skeletal maturation; however, owing to significant interindividual variability, it has traditionally been considered ineffective for assessing skeletal maturity.

However, our study demonstrates that once the humeral head ossification centre becomes visible—typically after 3 months of age—its longitudinal diameter shows a strong linear correlation with chronological age, supporting its potential use as a quantitative indicator in clinical practice. In addition, heteroscedasticity was observed in the regression residuals, particularly among younger female participants, reflecting greater variability in ossification centre size at early ages. This variability should be considered when interpreting regression-based estimates in very young infants.

The establishment of ± 2 SD reference ranges further enhances the clinical utility of this method. These ranges provide a quantifiable benchmark for identifying deviations from expected skeletal maturation. For example, in a 12-month-old boy, a measured longitudinal diameter of approximately 10 mm would fall within the ± 1 SD range in our reference data, indicating skeletal maturation appropriate for chronological age. Conversely, a substantially smaller or larger value—such as one falling outside the ± 2 SD band—could suggest delayed or advanced maturation, prompting further clinical evaluation. Such deviations may reflect underlying nutritional deficiency, endocrine dysfunction, or developmental disorders, whereas accelerated maturation may signal precocious puberty or other endocrine pathologies. As such, this method may serve as a practical screening tool for paediatricians to identify children requiring further evaluation or referral.

A major challenge in assessing skeletal maturation in children aged < 3 years is the limited number of ossification centres in the carpal bones, which makes classical hand-based methods ineffective.

In clinical settings, alternative methods for assessing skeletal maturation in children aged < 3 years have been proposed, such as those based on the knee joint, where ossification progresses more predictably. These include the use of radiographic standards, such as the Pyle and Hoerr atlas for the knee [3], or more recent approaches using deep learning models for knee radiographs [4]. Additionally, the Sontag method, which uses hemiskeleton radiographs, is also known [5]. However, these methods require additional X-ray imaging, thereby increasing radiation exposure and clinical workload. In contrast, our approach utilises routinely acquired chest radiographs, avoiding additional radiation and simplifying the assessment process.

Studies examining racial differences in bone age estimation based on the Greulich and Pyle atlas have shown that Asian and Hispanic children mature earlier than African American and Caucasian children. However, this difference is primarily evident in girls aged 10–13 years and boys aged 11–15 years, with no significant differences observed in early childhood [9]. This finding suggests that our method for children under 3 years may be applicable across different ethnic populations. Additionally, a study of Afghan children demonstrated that low total caloric intake and delayed skeletal maturation, as reflected by delayed bone age, indicate nutrition-related growth retardation [10]. While our reference ranges may require adjustment in regions where malnutrition is prevalent, this characteristic could make our method useful as a nutritional screening tool.

Previously, the timing of ossification centre appearance in the humeral head was considered highly variable among individuals, limiting its use for assessing skeletal maturation. Notably, our findings indicate that once the ossification centres become visible—typically after 3 months of age—their size strongly correlates with chronological age, supporting their use as a developmental indicator. This represents a significant advancement in the assessment of early skeletal maturation, as, to the best of our knowledge, no prior studies have investigated the use of humeral head ossification centres for this purpose. In contrast, studies on adult populations have investigated the relationship between the morphometric measurements of the proximal humerus and chronological age [11]. However, these studies reported no correlation between the surface area of atrophy in the ossification centres of the humeral head obtained from cadaveric specimens and the actual age. Conversely, our findings clearly demonstrated a strong correlation between the longitudinal diameter of the ossification centres of the humeral head and age in children.

In our study, the ossification centres of the humeral head were not visible in some cases within the 0- to 2-month age group. Ogdon et al. reported that the ossification centre of the humeral head was evident on radiography between 2 and 3 months of life [12]. In magnetic resonance imaging studies, Kwong et al. reported the appearance of the ossification centre of the humeral head between 2 and 4 months of age [13]. Our findings agree with those of previous studies, demonstrating that the right- or left-sided ossification centre of the humeral head was consistently visible after 3 months of age in nearly all cases.

The establishment of ± 2 SD ranges for the longitudinal diameter of the ossification centres is another significant contribution of this study. Measurements exceeding these ranges may indicate accelerated or delayed bone maturation relative to chronological age. Bone age assessment is a crucial tool for detecting developmental and growth abnormalities in children. Certain congenital anomalies and genetic disorders can potentially cause delayed or accelerated skeletal maturation [14, 15]. Early detection of these conditions can facilitate the timely initiation of appropriate treatment. Nutritional status is reflected in bone age, as demonstrated by the Infancy-Childhood-Puberty model [16]. Chronic malnutrition may lead to delayed skeletal maturation, potentially serving as an indicator of the need for nutritional intervention [17]. Abuse and neglect can affect growth and development, with delayed skeletal maturation, potentially suggesting an inadequate nurturing environment [2]. Therefore, evaluation of accelerated or delayed bone maturation using this method can lead to further examinations or referrals to specialists. This approach enhances our ability to identify and address various factors that affect paediatric growth and development, including genetic disorders, nutritional deficiencies, and potential cases of child neglect or abuse.

Another strength of this study is the simplicity and reliability of the measurement process. The longitudinal diameters of the ossification centres of the humeral head are easy to measure and do not require specialised training. Despite the varying levels of experience among the readers involved, interobserver variability was minimal, underscoring the robustness of this method. Additionally, chest radiography is a routine and cost-effective diagnostic tool, further supporting the clinical utility of this approach for assessing skeletal maturation in children aged < 3 years. Given that the humeral head is often included in routine chest radiographs, this method requires no additional imaging, thereby avoiding extra radiation exposure—a significant advantage in paediatric care that aligns with the as low as reasonably achievable principle [18].

To further enhance clinical applicability, we derived simplified estimation formulas based on the average regression coefficients observed across the three readers. These allow clinicians to quickly approximate the expected ossification centre size from the child’s age in months without referring to full scatterplots or standard deviation curves:

For males: longitudinal diameter (mm) ≈ 0.5 × age in months + 4.

For females: longitudinal diameter (mm) ≈ 0.6 × age in months + 3.

In addition, to facilitate clinical interpretation, the inverse equations were also provided to estimate age directly from measured ossification centre size:

For males: age (months) = 2 × (longitudinal diameter (mm) − 4).

For females: age (months) = 1.7 × (longitudinal diameter (mm) − 3).

These formulas offer a practical tool for rapid assessments in clinical settings. Conventional hand-based bone age assessment using the Greulich and Pyle Atlas is influenced by clinical experience [19]. However, our approach demonstrated high interobserver agreement and can allow for reliable assessment, regardless of the examiner’s clinical experience.

This study did not include direct comparison with established hand-based or knee-based skeletal maturity assessment methods. Alternative methods such as knee radiographs would have required additional radiation exposure and were not available in our retrospective cohort, making direct comparison methodologically challenging. Therefore, our proposed method should be considered as a supplementary screening tool that leverages routinely available chest radiographs. This approach is not intended to replace conventional hand and wrist radiographs, which remain the standard low-radiation method for evaluating growth and maturation disorders. Instead, it provides complementary information from routinely obtained chest radiographs without additional radiation or imaging procedures. In future work, prospective studies should be conducted to validate the present approach against established hand and wrist–based methods, including the Greulich–Pyle (GP) and Tanner–Whitehouse (TW) systems, as well as automated deep learning–based techniques such as BoneXpert. Such comparisons will be essential to confirm the accuracy, reproducibility, and clinical relevance of our method and to position it alongside existing gold-standard approaches for paediatric skeletal maturity assessment. Future prospective validation studies should be conducted to compare our method with knee-based techniques (such as the Pyle-Hoerr atlas) when both imaging modalities are clinically indicated.

However, this study has some limitations. First, the study population consisted exclusively of East Asian children, which may limit the generalisability of the findings. Future multicentre studies across diverse populations are necessary to validate the applicability of the proposed reference ranges. Second, although our sample size was sufficient for initial modelling, larger datasets would improve statistical robustness.

Third, no adjustment was made for body size or anthropometric parameters, which may influence the apparent dimensions of the ossification centre, particularly in younger infants. In addition, although overtly malrotated radiographs were excluded, subtle radiographic rotation or obliquity was not formally corrected for, and this may have introduced measurement variability. All radiographs were acquired under standardised imaging protocols, and calibration testing confirmed consistent image scaling across the devices; therefore, the influence of magnification variability on our measurements was considered negligible. However, as all radiographs were obtained within a single institution, potential inter-institutional or multi-centre imaging variability could not be assessed. Future multi-centre studies incorporating calibration markers or standard reference objects may help improve measurement consistency. Finally, the exclusion of children with metabolic or skeletal disorders means further studies are required to assess the method’s diagnostic value in pathological contexts. Furthermore, although this study demonstrated excellent interobserver reliability (ICC = 0.96, p < 0.001), intraobserver validation over time was not performed. Future studies should include blinded re-measurement to confirm the temporal stability of this measurement approach.

In conclusion, we propose a novel, practical, and reproducible method for assessing skeletal maturation and approximating chronological age in children aged < 3 years using humeral head ossification on routine chest radiographs. While the findings suggest that this technique may serve as a useful adjunct in paediatric care—particularly when traditional hand-based methods are not feasible—its clinical applicability should be interpreted with caution given the retrospective, single-centre design of the study and the methodological limitations described. Future prospective multicentre studies will be essential to validate these findings and to confirm the generalisability of this approach across diverse populations before wider clinical implementation.

## Supplementary Information

Below is the link to the electronic supplementary material.

Supplementary file1 (PDF 270 KB)

## Data Availability

The data that support the findings of this study are available on request from the corresponding author, Hiromi Edo (miki3suntree@yahoo.co.jp). The data are not publicly available due to ethical restrictions approved by the Ethics Committee of the National Defense Medical College, which permit handling of the data only within the institution. However, de-identified data can be provided to researchers subject to approval. Compliance with ethical standards: This study complied with the ethical guidelines of the Declaration of Helsinki and was approved by the ethics committee of our institution (National Defense Medical College, 5036). The requirement for written informed consent was waived because of the retrospective nature of the study, and patient records were anonymously analyzed.

## Abbreviations

ICC: Intraclass correlation coefficient
R^2^: Coefficient of determination
SD: Standard deviation
