# Supplementary material for: A practical tip for estimating skeletal maturation in children aged < 3 years using humeral ossification on chest radiographs: A retrospective study
Source: Eur J Pediatr. 2026 Jan 29;185(2):111. doi: 10.1007/s00431-026-06760-6 (PMC12855226; doi:10.1007/s00431-026-06760-6)

A Practical Tip for Estimating Skeletal Maturation in Children Aged < 3 Years Using Humeral Ossification on Chest Radiographs

European Journal of Pediatrics

Tomohiro Tsuru[1*] (0009-0006-6913-0868)**, Shota Inoue**[1*] (0000-0002-4854-4897)**, Hiromi Edo**[2*] (0000-0002-9602-2700), **Shuichi Suzuki**[1], **Kohsuke Imai**[1] (0000-0003-2132-8403), **Taiki Nozaki**[2] (0000-0002-1922-4085), **Hiroshi Shinmoto**[2] (0000-0002-3474-2913)

[1]Department of Pediatrics, National Defense Medical College, Saitama, Japan

[2]Department of Radiology, National Defense Medical College, Saitama, Japan

[*]These authors contributed equally to this work.

Corresponding author's email: E-mail: miki3suntree@yahoo.co.jp

Supplementary Material

Online Resource 1 (Table S1). Visibility of humeral head ossification centres by age group and sex.

Online Resource 2 (Table S2). Sex-specific linear regression coefficients for each reader.

Online Resource 3 (Fig. S1). Comparison of ossification centre diameter by arm position.

Table S1. Visibility of humeral head ossification centres by age group and sex

Male				Female			
Age Group (Months)	n	Right (%)	Left (%)	Age Group (Months)	n	Right (%)	Left (%)
0-2	20	14 (70)	15 (75)	0-2	15	13 (86.6)	10 (66.6)
3-5	11	11 (100)	10 (90.9)	3-5	2	2 (100)	2 (100)
6-8	9	9 (100)	9 (100)	6-8	3	3 (100)	3 (100)
9-11	6	6 (100)	6 (100)	9-11	9	9 (100)	9 (100)
12-14	10	10 (100)	9 (90)	12-14	7	7 (100)	7 (100)
15-17	12	12 (100)	12 (100)	15-17	9	8 (88.8)	9 (100)
18-20	9	8 (88.8)	9 (100)	18-20	11	10 (90.9)	11 (100)
21-23	8	8 (100)	8 (100)	21-23	4	3 (75)	4 (100)
24-26	2	2 (100)	2 (100)	24-26	7	7 (100)	7 (100)
27-29	6	6 (100)	6 (100)	27-29	9	6 (66.6)	8 (88.8)
30-32	3	2 (66.6)	3 (100)	30-32	2	2 (100)	2 (100)
33-35	9	6 (66.6)	9 (100)	33-35	4	4 (100)	4 (100)

Note: In the 0–2-month group, 8 cases (5 males and 3 females) had no visible ossification centres bilaterally and were therefore treated as 0 mm in the analysis. In all other age groups, at least one humeral head ossification centre was visible in every case, enabling diameter measurement.

Table S2. Sex-specific linear regression coefficients for the relationship between age and humeral head ossification centre diameter.

Linear regression models were fitted separately for males and females, with chronological age (months) as the independent variable and the longitudinal diameter (mm) of the humeral head ossification centre as the dependent variable. Slopes and intercepts are reported for each reader with corresponding 95% confidence intervals (CI).

Male		
**Reader**	**Slope (95% CI)**	**Intercept (95% CI)**
Reader A	0.50 (0.45–0.54)	4.06 (3.30–4.82)
Reader B	0.50 (0.45–0.55)	4.18 (3.33–5.03)
Reader C	0.47 (0.43–0.52)	3.89 (3.12–4.67)

Female		
**Reader**	**Slope (95% CI)**	**Intercept (95% CI)**
Reader A	0.64 (0.58–0.70)	2.66 (1.49–3.84)
Reader B	0.58 (0.51–0.65)	3.63 (2.33–4.94)
Reader C	0.58 (0.52–0.63)	2.90 (1.81–3.99)

Table S3. Simplified formulas for clinical estimation of bone age

Sex	Formula for expected diameter	Formula for estimated age
Male	Diameter (mm) = 0.5 × age (months) + 4	Age (months) = 2 × (Diameter － 4)
Female	Diameter (mm) = 0.6 × age (months) + 3	Age (months) = 1.7 × (Diameter － 3)

Simplified sex-specific formulas derived from the averaged linear regression coefficients of the three readers. These formulas allow estimation of the expected humeral head ossification centre diameter from chronological age, and vice versa, for clinical screening purposes.

4

Fig. S1 Comparison of the longitudinal diameter of epiphyseal ossification centers by arm position

Scatterplots of the longitudinal diameter of the humeral head ossification centers by age in male (left) and female (right), based on arm position during chest radiography. Open circles represent cases with the arms in the raised position, and black squares represent those with the arms in the lowered position. No apparent difference in measurement distribution is observed between the two groups, supporting the conclusion that arm position did not significantly affect the measurement results.

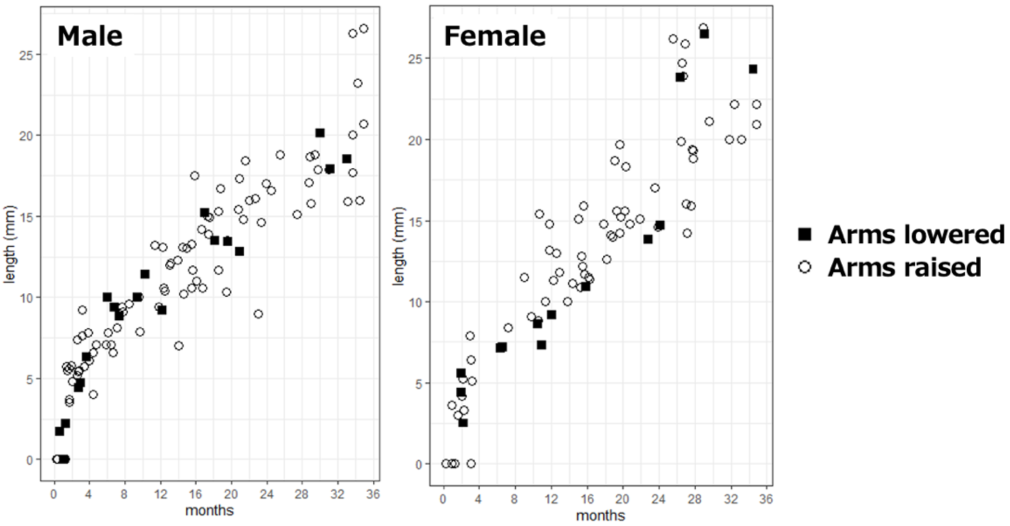

Supplement: Supplementary file 1 — Supplementary file1 (PDF 270 KB) [file 431_2026_6760_MOESM1_ESM.pdf]
